# Carotid Intima Media Thickness as a Measure of Cardiovascular Disease Burden in Nigerian Africans with Hypertension and Diabetes Mellitus

**DOI:** 10.1155/2011/327171

**Published:** 2011-05-23

**Authors:** Basil N. Okeahialam, Benjamin A. Alonge, Stephen D. Pam, Fabian H. Puepet

**Affiliations:** ^1^Department of Medicine, Jos University Teaching Hospital, PMB 2076, Jos, Plateau State, Nigeria; ^2^Department of Radiology, Jos University Teaching Hospital, PMB 2076, Jos, Plateau State, Nigeria

## Abstract

As part of a larger study of cardiovascular risk factors in nonhypertensive type 2 diabetes patients, we subjected a cohort of diabetics to B mode ultrasonography of the carotid artery to measure the intima media thickness (IMT) and compared it with values in hypertensives and apparently normal controls matched reasonably for gender and age. All groups were comparable in terms of age and gender representation. The mean (SD) of carotid IMT right and left was 0.94 mm (0.12), 0.94 mm (0.16); 0.93 mm (0.21), 0.93 mm (0.15); 0.91 mm (0.17), 0.91 mm (0.13) for diabetic, hypertensive, and normal groups, respectively. There was a nonsignificant tendency to raised IMT for the disease groups from the normal ones. Diabetic and hypertensive Nigerians are equally burdened by cardiovascular disease risk factors. Apparently normal subjects have a reasonable degree of burden suggesting the need to evaluate them for other traditional and emerging risk factors.

## 1. Introduction

Diabetes mellitus (DM) currently affects 6% of the world's adults, a figure expected to double in the western world and triple in Africa and Asia by 2010 [1]. In Nigeria, the national prevalence is estimated at 2.2% [2]. In Jos, Puepet reported a community prevalence of 3.3% [3]. Clinical and epidemiological studies have clearly demonstrated that diabetics face increased cardiovascular disease morbidity and mortality [4] with 3 out of 4 of them dying with cardiovascular complications [5]. More than half of all diabetic deaths are accounted for by atherosclerotic disease [6]. Hypertension on its own is the greatest cause of death from cardiovascular atherosclerotic disease, a role that is already firmly established [7]. Its prevalence in Nigeria ranges from 17 to 20% [8], and approximately 15 to 37% of the world's adult population is afflicted [9].

Intima media thickness (IMT), a measure of atherosclerotic vascular disease, is considered as a comprehensive picture of all alterations caused by multiple risk factors over time on the arterial walls [10]. It can therefore be described as a robust indicator of vascular risk. Traditional and some emerging cardiovascular disease risk factors have shown a positive association with IMT in epidemiological studies of patients and general population [11–13]. It can easily be measured especially at the carotids by B mode ultrasonography, a relatively simple way representing a safe, precise, and reproducible measure [14]. In resource-limited settings like ours, cost of equipment and dearth of skilled personnel restrict the use of this investigative tool in clinical practice. Whatever the method or site of measurement including the distal common carotid far wall IMT, increased IMT has been shown to be a powerful predictor of coronary and cerebrovascular complication [15]. It has therefore been proposed as a noninvasive measure of cardiovascular disease burden in adults [16].

With the acquisition of B mode ultrasound facility in our centre and a radiologist skilled in such measurements, we decided to compare the atherosclerotic burden of DM with hypertension (the flagship of atherosclerotic vascular disease) and a nondiabetic, nonhypertensive apparently normal control group. The findings are expected to galvanise clinicians towards aggressive life style and therapeutic intervention to stem the tide of rising cardiovascular diseases.

## 2. Methodology

This was part of a larger study of cardiovascular risk factors in nonhypertensive type 2 DM in Jos University Teaching Hospital, Jos, carried out between November 2005 and May 2006. The minimum sample size calculated for the study was 34, but it was doubled and rounded up to 70 for each group [17]. The hospital ethics committee approved the study, and informed consent was got from all participating patients and subjects. Group A was made up of consenting consecutive type 2 DM patients ≥30 years who were attending and receiving treatment at the Diabetes and Medical Out Patients clinics. They were 70 and had no concomitant hypertension. Group B was made up of 70 hypertensive patients matched as much as possible for age and sex with the diabetics, from the Cardiology and Medical Out Patient clinics where they were undergoing treatment. Group C was made up of 71 age- and sex-matched apparently normal subjects recruited from the General Out Patient clinics where they presented for minor ailments only. They had neither hypertension nor diabetes confirmed with physical examination and relevant laboratory investigation. They all underwent carotid ultrasonography in the Radiology Department. All measurements were performed by a single experienced radiologist (SDP) who was blind to the clinical status of the patients and subjects. This was to reduce interobserver error.

The examination was done using a duplex scanner (HP Sonos 1500—Hewlett-Packard) with a 7.5 MHz linear array transducer. The patients and subjects were laid supine, and both common carotids (left and right) were scanned with their heads tilted to the opposite side and neck slightly extended. The IMT was defined as the distance between the leading edge of the luminal echo to the leading edge of the adventitia of the media. This measurement was taken at a site of 1.0 cm proximal to the carotid bulb.

## 3. Results

The 70 diabetics (Group A) were made up of 36 females and 34 males, while the 70 hypertensives (Group B) consisted of 37 females and 33 males. The 71 control subjects (Group C) consisted of 36 females and 35 males. There was no statistically significant difference in proportion between the sexes in the groups.

The age range and means (SD) of the patients were 30 to 71 years and 51.2 (10.63), 30 to 85 years and 49.8 (12.03), and 30 to 82 years and 49.7 (11.02), respectively, for groups A, B, and C. There was also no statistically significant difference between them.

The mean carotid intima medial thickness (CIMT) of the patients is shown in Table 1. There was a tendency towards increased CIMT from normal for the hypertensives and the diabetics, the latter more than the former. The difference did not attain statistical significance, however.

Using values in excess of 0.91 mm to define carotid atherosclerosis since mean CIMT for our normals here was 0.91 mm (in the absence of normative values for Nigerian Africans), prevalence of carotid atherosclerosis was 47.5% for diabetics, 48.9% for hypertensives, and 36.5% for normal controls. Prevalence rates of atherosclerosis for both disease states were similar, and they were significantly higher than the apparently normal group (*P* < .001).

## 4. Discussion

Although CIMT is not yet routinely measured in clinical practice, its predictive value regarding cardiovascular complications has been established, giving it a potential role in future for cardiovascular disease risk stratification and primary prevention [18]. This study has shown an increased tendency to atherosclerotic cardiovascular disease in both DM and hypertension going by their CIMT values. The similarity in values for both sides would suggest that in practice, measuring either side would suffice.

The increase in CIMT as an index of atherosclerosis occurs in conjunction with other cardiovascular risk factors [19]. It would appear that going by the results in this study, the cases of DM had a greater cardiovascular disease burden. Their burden of cardiovascular disease risk factors surpassed that of Hypertension in most cases especially dyslipidaemias, waist hip ratio, cigarette smoking, alcohol abuse, physical inactivity, and serum creatinine (results not shown). This is in accord with the work of Weiss and Sumpio [20], where it was found that diabetes caused higher morbidity and mortality from vascular diseases. Modifiable cardiovascular risk factors in DM should therefore be treated aggressively to reduce complications arising from cardiovascular diseases.

Of all traditional risk factors for cardiovascular diseases, hypertension is said to have the greatest effect on IMT via medial hypertrophy, a process specifically related to the disease [21]. The results here would suggest otherwise until looked at from the carotid atherosclerosis view point, where hypertension marginally overtook DM in prevalence rate.

Our findings are consistent with the experiences of Adaikkappan et al. [22] and Dikanovic et al. [23] where CIMT of hypertensives and diabetics, respectively, were significantly greater than normal controls. However, when the mean CIMT value of 0.91 mm for both sides in this study is compared with the normal CIMT of 0.74 mm for the right and 0.72 mm for the left carotids in the work of Adaikkappan et al. [22], it becomes obvious that apparently normal Nigerian Africans living on the Jos, Plateau have higher CIMT values and by extension higher cardiovascular disease risk. This re-enforces the need for normative values for each region in evaluating risk factors for cardiovascular diseases. Sorof et al. [24], given their findings of high CIMT correlating positively with left ventricular hypertrophy, speculated that CIMT represents the same adaptive process to that responsible for development of hypertension. Africans are known to have a greater tendency to left ventricular hypertrophy [25], which has been thought to be either genetic or environmental, even before the development of hypertension. Our higher average IMT values may have to do with higher prevalences of certain infections like hepatitis B [26], hepatitis C [27], and human immunodeficiency virus infection [28]. These have been shown to be associated with increased IMT [29]. The role of underlying local and systemic inflammation in response to bacterial infection and other inflammatory conditions with their proinflammatory cytokines in atherogenesis [30] is also relevant. Infections of all variety are still rife in our environment. Finally, psychological stress levels which have been reported more in Africans [31] and are associated with higher CIMT [32] complete the triad of environmental putative mechanisms for atherosclerosis and high IMT. We did not comprehensively screen the study subjects for these and other new and emerging cardiovascular risk factors. Consequently, we can not say if and how much of these are operating.

## 5. Conclusion

Carotid IMT as documented in many studies in the western world also mirrors cardiovascular disease burden in DM and hypertension in Nigerian Africans. Apparently normal nondiabetic, nonhypertensive Nigerians also have a high cardiovascular disease burden going by their CIMT values. This calls for exploration of burden of newer risk factors like chronic periodontitis and poor oral health [33] as well as particulate air pollutants [34] in our environment. The role of the staple potato diet eaten in this environment especially fried deserves consideration. Chronic intake of potato chips in humans with its high acrylamide content is known to induce a proinflammatory state, a risk factor for progression of atherosclerosis [35]. Not withstanding the foregoing, CIMT when determined in individual cases could be a guide to how aggressively modifiable atherosclerotic risk factors should be sought and addressed, apart from its role in prognostication.

## Figures and Tables

**Table 1:** Mean (SD) carotid intima media thickness in study population.

Groups	R. CIMT	L. CIMT
Diabetics (A)	0.94 mm (0.12)	0.94 mm (0.16)
Hypertensives (B)	0.93 mm (0.21)	0.93 mm (0.15)
Normals (C)	0.91 mm (0.17)	0.91 mm (0.13)
